# Seroprevalence of visceral leishmaniasis and its associated factors among asymptomatic pastoral community of Dire District, Borena zone, Oromia Region, Ethiopia

**DOI:** 10.3389/fpubh.2022.917536

**Published:** 2022-11-21

**Authors:** Haile Ketema, Fitsum Weldegebreal, Abdella Gemechu, Tesfaye Gobena

**Affiliations:** ^1^Borena Zone Health Department Office, Malaria and NTD, Yabelo, Ethiopia; ^2^School of Medical Laboratory Sciences, College of Health and Medical Sciences, Haramaya University, Harar, Ethiopia; ^3^Department of Environmental Health Sciences, College of Health and Medical Sciences, Haramaya University, Harar, Ethiopia

**Keywords:** seroprevalence, associated factors, *Leishmania donovani*, Dire District, Ethiopia, visceral leishmaniasis

## Abstract

Visceral leishmaniasis (VL) is a vector-borne protozoan neglected tropical disease. In some parts of Ethiopia, it is a public health problem and its main causative agent is *the Leishmania donovani complex*. The objective of the study was to determine the seroprevalence of VL and factors associated among the asymptomatic pastoral community of Dire District, Borena Zone, Oromia Region, Ethiopia. A community-based study was conducted among 432 pastoralist communities from June to July 2021. A systematic random sampling method was used to select households. Pretested structured questionnaires and face-to-face interviews were used to collect data. A single finger-prick blood sample was collected and tested for *Leishmania donovani complex* using an immune-chromatographic test (rk39-ICT). A logistic regression model was used to assess factors associated with VL infection and a *p*-value of < 0.05 was considered statistically significant. A total of 432 study participants were included (their mean age was 26.69) and 218 (50.5%) were females. The overall seroprevalence of VL was 33/432(7.6%) (95%CI: 5.32–15.60). Sero-prevalence was significantly associated with high family size (>5) (adjusted odds ratios (AOR) = 5.134; 95% CI: 2.032–9.748), sleeping or/and staying under acacia tree (AOR = 2.984; 95%CI = 1.074–8.288), presence of cracked house walls (AOR = 1.801; 95%CI: 1.026–4.926), presence of termite hills (AOR = 1.938; 95%CL: 1.002–7.050), availability of water points (AOR = 3.893; 95%CI: 1.034–7.426) and presence of domestic animals (AOR = 2.124; 95% CI: 2.341–5.108). It is recommended that community awareness on the transmission and prevention methods of *Leishmania donovani complex* and taking appropriate interventions on the identified factors play a greater role to prevent and control infection in the area. Further investigation is also needed to characterize the pathogens and risk factors and tackle the problem.

## Introduction

Leishmaniases are a set of diseases caused by near to 22 species of the protozoan genus *Leishmania* and are transmitted between humans and other mammalian hosts (1). Clinically, there are two major forms of leishmaniasis, cutaneous leishmaniasis (CL) and visceral leishmaniasis (VL). VL is the severe form of leishmaniasis, fatal if not treated, and is among tropical neglected protozoan diseases and caused by *Leishmania donovani complex* (*L. donovani and L. infantum;* are the causative agents of VL in Ethiopia) (2, 3) and *Leishmania donovani* is the principal causative agent (4).

Infection and transmission of VL are through the bite of the female phlebotomine sand-fly and inoculation of the infective stage of the parasite (5). Humans, hyraxes, and domestic dogs are the most commonly infected hosts, and they can also be potential reservoirs (6, 7). Domestic dogs might be the most important reservoir of both *L. donovani* and *L. infantum* in eastern Africa (6, 8). However, the transmission of *L. donovani* is generally thought to be anthroponotic in the East African region, and in Ethiopia, it is thought to be partially zoonotic and anthroponotic, the nature of which varies with geographical areas (8, 9). Thus, the decisive reservoir of VL in East Africa and Ethiopia remains to be determined and wellstudied. In addition, multiple factors like seasonal migration, biological, environmental, and areas of lower socioeconomic status are factors that contribute to disease transmission in the area (10). The presence of sandflies and gorges in the area, open waste disposal, the presence of a reservoir host, and asymptomatic cases are important risk factors for the spread of the disease (11).

Globally, the disease affects ~350 million people in 98 countries; where most of them are in developing regions. However, ~90% of the global VL burden is attributed to a few countries: Bangladesh, Brazil, India, Ethiopia, Sudan, and South Sudan (12). The report indicated that there are many foci of VL infection in eastern African countries (13, 14).

In Ethiopia, VL is predominantly found in the lowlands with varying degrees of endemicity and the vectors responsible for disease transmission are *Phlebotomus martini, Phlebotomus celiae*, and *Phlebotomus orientalis* (15). Approximately 3.2 million people in the population are at risk of VL and north-eastern, north-western, western, and south-eastern parts of Ethiopia are geographically suitable for disease transmission (16). Annually, 3,700–4,000 estimated VL cases occur in Ethiopia (17). The northern and northwest parts of Ethiopia have the highest burden, which accounts for nearly 30–40% of the total number of Ethiopian VL patients; of which around 30% of VL patients are malnourished and co-infected with HIV (18). While the southern foci (the south-western savannah and the south-eastern semi-arid lowlands) account for ~20% of the total VL burden in Ethiopia (13).

According to the Oromia Regional Health Bureau report, VL is endemic in Borena, Guji, and Bale zone (19). The Borena zone started to report VL cases in 2012 from Aero, Dire, and Miyo Districts. Currently, the problems could be extended to other districts but still more cases were from Dire districts and as a result, outbreaks of VL occurred in 2020 (20). In a nutshell, limited studies have determined the magnitude of VL due to *the Leishmania donovani complex* and its associated factors in Ethiopia. Moreover, no studies have been conducted to determine the status of seroprevalence and associated factors with VL in the study area. Thus, this study was conducted to determine the seroprevalence of *the Leishmania donovani* and factors associated with the asymptomatic pastoral community of the Dire District, Borena Zone, and Oromia region in Ethiopia.

## Methods

### Study area and period

**Figure 1:**
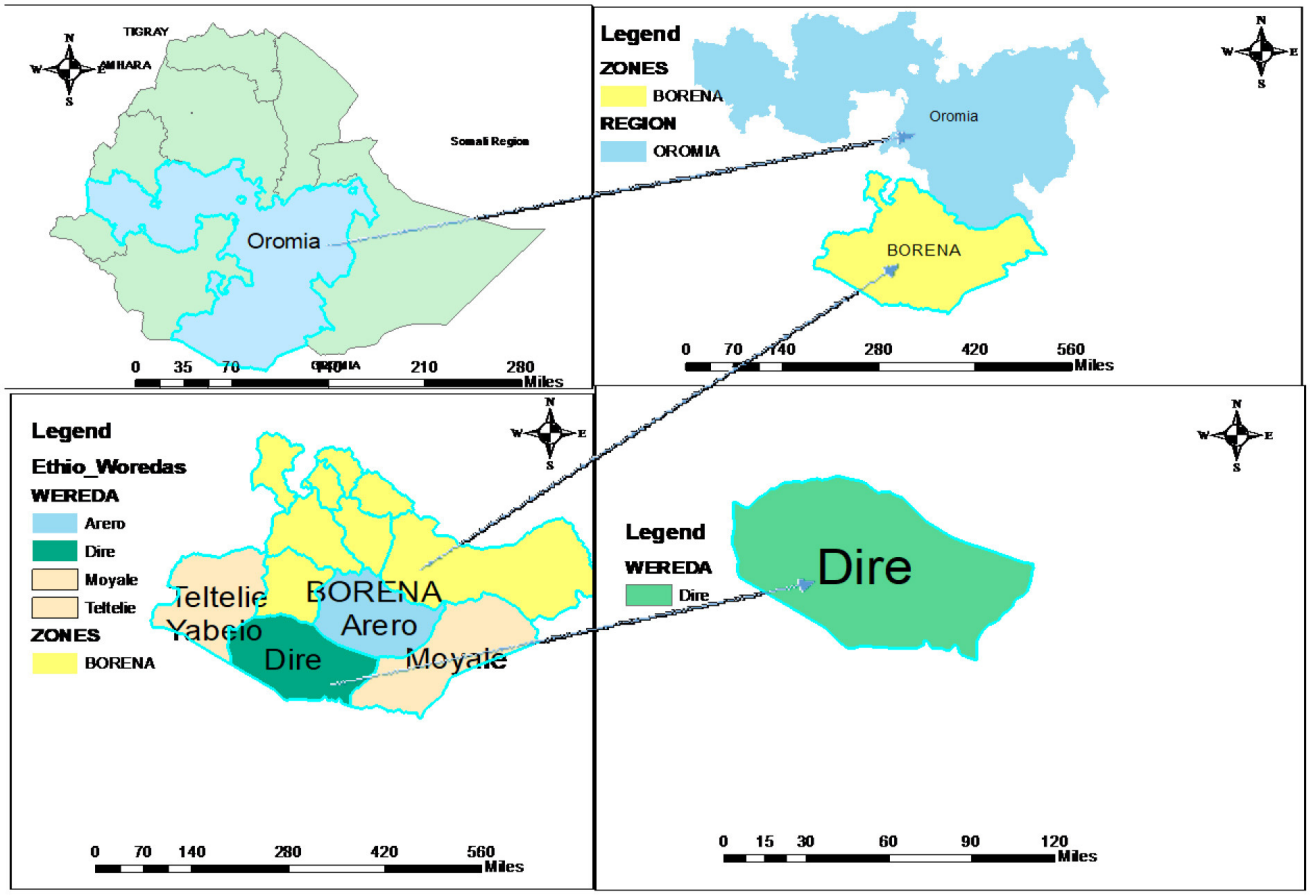
Map of Dire District, Borena zone, Oromia Region, Ethiopia 2021.

The study was carried out in Dire District in the Borena Zone. The Dire District is one of the 13 Districts in the administrative divisions of the Borena zone and is located 100 km from Yabalo town, the capital of the Borena zone, and 675 km from Addis Ababa. The woreda has 13 kebeles (smallest administrative unit) and one administrative town (Figure 1). According to the 2021 projection, the district has about 53,376 populations. Pastoralists remain the dominant livelihood system of the people, though some of the community members are gradually tending to Agro-pastoralist (livestock and crop production) livelihoods as a coping mechanism. The average rainfall varies from 300 mm in the low area to more than 740 mm in higher altitudes. The district has 1 hospital, 5 health centers, 17 health posts, and 6 private clinics, and the potential health service coverage is estimated to be 100% (21). The study was conducted from June to July 2021.

### Study design and population

A community-based cross-sectional study design was conducted. The selected household (HH) members of the kebeles who fulfill the inclusion criteria were included in this study. Visceral leishmaniasis confirmed cases and treatment during data collection and individuals who had a previous history of VL disease were excluded.

### Sample size determination

The sample size was determined using Epi-info version 7 software and the presence of animals in the yards with an exposed outcome of 41.1% (22), a confidence level of 95%, a margin of error of 5%, power of 80%, and the ratio of exposed to unexposed 1:1 were considered. In addition, the multistage sampling technique was used after adjusting for the design effect by a factor of 1.5 and 10% of the non-response rate; the final sample size became 432.

### Sampling procedure

**Figure 2:**
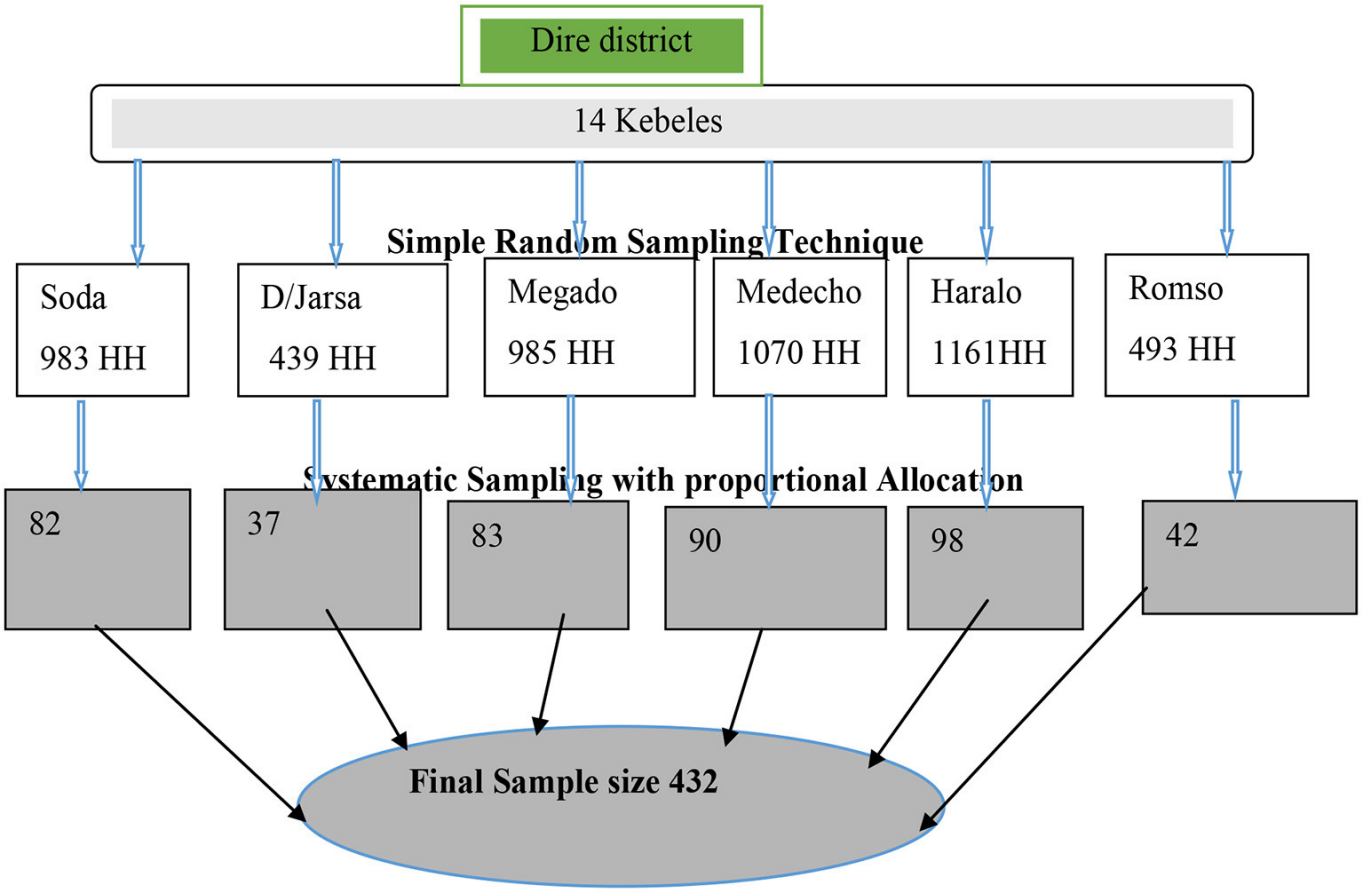
Schematic presentation of sampling procedure.

Kebeles and households (HHs) were used as primary and secondary sampling units, respectively to reach the study participants of the multistage sampling technique. From a total of 14 kebeles, 6 kebeles were selected using a simple random sampling method. The allocation of the sample to the kebeles was undertaken proportionally based on the number of HHs in the selected kebeles (Figure 2). The list of HHs in each of the study kebeles was obtained from Health posts (Health Extension workers). The sampling interval was established based on the total number of households in the kebeles and the 12^th^ HH was selected from each kebele. Households were substituted with the nearest neighbor in cases of refusal to participate or if the HH was not inhabited. The selected HHs were given a unique identification number.

### Data collection instruments and procedure

Four trained data collectors with college-level diploma graduates, two supervisors, and two Laboratory professionals from the Leishmaniasis treatment center at Yabalo Hospital and Mega Health center participated in the study. All data collectors were fluent speakers of the local language. Data were collected using a pre-structured questionnaire adopted from previous literature (22–24). Initially, the questionnaire was developed in English and translated into Afan Oromo and then back to English to check for its consistency.

Data were collected by face-to-face interviews using a structured Afan Oromo version questionnaire. The data and blood samples were collected from one person using the lottery method approach and were selected from any of the family members of the selected HHs. If a selected individual was < 18 years old the questionnaire-related data were collected from parents whose age was >18 years but a blood sample was collected from the selected person themself. If the selected person was adult individual both data and the blood sample were collected at the same time.

### Sample collection and processing

Blood samples were collected from an individual selected from HH members using lottery methods. During sample collection, each participant's finger was cleaned using alcohol and cotton. A single finger prick blood specimen (8–12 μL) was collected and added to the absorbent pad well with 150 μl (2–3 drops) of the chase buffer provided with the kit (25, 26). Following the manufacturer's instructions, a serological diagnosis of *L. donovani complex* was then performed by using the rk39 ICT (IT LEISH, Bio-Rad, France) (27). Finally, the results were read after 10–20 min and interpreted as: reactive when both control and test lines appear; non-reactive when only the control line appears; invalid when no control line appears. Using whole blood samples, the sensitivity and specificity of the rK39 immunochromatographic test used for this study were 99 and 100% respectively based on the manufacturer's instruction.

### Data analysis

Data entries were made using EpiData 3.1 version and then exported to Statistical Package for Social Science (SPSS) V-20 computer software for analysis. Descriptive statistic was used to summarize data. Independent variables with a *p*-value of 0.20 or less in the bivariable logistic regression were included in the multivariable logistic regression and each covariate effect was adjusted. Variables with a *p*-value ≤ 0.05 were considered for the significant association.

### Data quality control

Designing proper data collection tools, and training for data collectors, laboratory professionals, and supervisors were measures taken to assure data quality. Before the actual data collection, the questionnaire was tested on 5% of the respondents in a neighboring district. During data collection, questionnaires were reviewed and checked for completeness, accuracy, and consistency on the daily basis. The VL serological test kit (the rk39) was checked for expiration date and batch before using the test and its standard operating procedures were strictly followed.

### Ethical consideration

Ethical clearance was obtained from Haramaya University, College of Health and Medical Sciences Institutional Health Research Ethics Review Committee (IHRERC) (Ref. No. IHRERC/078/2021). A letter of support was obtained from Borena Zone Administration and Zonal health office, as well as the Dire district Administrations and Health Offices. Moreover, study participants were informed about the purpose of the study, and their consent was obtained before data collection. For children and adolescents aged < 18 years, informed voluntary written signed consent was obtained from their parent or legal guardian. In addition, oral assent was obtained from the child and/or adolescents aged < 18 years. Adolescents aged 18 years and adults were provided informed voluntary written and signed consent by themselves. All informed consent documents were provided in the local language and the confidentiality of the study participants was kept.

## Results

### Socio-demographic characteristics

A total of 432 study participants were included. Their mean age was 26.69 (standard deviation (SD) = ±7.8) years (age range: 1 to 85 years) and 218 (50.5%) were female. The highest proportion of the age group was 10–19 years (31.5%), Muslim religion followers (47%), illiterate (49.3%), and married (61.1%). One hundred eighty-five (42.8%) of the HHs had an estimated family income between 500 and 1,000 Ethiopian Birr and 40.0% of the household's family size ranged from 3 to 5 (Table 1).

**Table 1:** Socio demographic characteristics of study participants of Dire District, Borena zone, Oromia Region, Ethiopia, 2021 (*n* = 432).

**Characteristics**	**Categories**	**Number**	**Percentage**
**Sex**	Male	214	49.5
	Female	218	50.5
**Age (in years)**	1–9	80	18.5
	10–19	136	31.5
	20–29	104	24.1
	≥30	112	25.9
**Marital status**	Single	151	35.0
	Married	264	61.1
	Divorced	9	2.1
	Widowed	8	1.8
**Occupation**	Cattle keeping	188	43.5
	Students	128	29.6
	Trading/skilled	116	26.9
	job/Merchant		
**Education**	Illiterate	213	49.3
	Read and write	137	31.7
	Elementary and above	82	19.0
**Religion**	Muslim	203	47.1
	Wakefata	192	44.4
	Catholic	21	4.8
	Protestant	10	2.3
	Orthodox	6	1.4
**Ethnic group**	Oromo	422	97.7
	Others**	10	2.3
**Family monthly**	< 500	117	27.1
**income (in Birr)**	501–999	185	42.8
	≥1,000	130	30.1
**Family size**	1–2	158	36.6
	3–5	173	40.0
	>5	101	23.4

**Amhara, gurage, tigray and kembata.

### Behavioral characteristics

Among the study participants about 20.1, 66.2, 48.6, 55.1, 37, and 30.3% were from a family having a history of infection with VL, have travel history to other VL endemic areas, know about the prevention of VL, have sleep on the floor/ground, have slept/stayed day and night under an acacia tree, and know about the transmission and control of VL, respectively (Table 2).

**Table 2:** Behavioral related factors among study participants of Dire District, Borena zone, Oromia Region, Ethiopia, 2021 (*n* = 432).

**Characteristics**	**Categories**	**Number**	**Percent**
Travel history to other VL endemic areas	Yes	286	66.2
	No	146	37.8
Family history of infection with VL	Yes	87	20.1
	No	345	79.9
Use bed net	Yes	87	21.1
	No	345	79.9
Sleeping/staying at day or/and night under Acacia	Yes	160	37
	No	272	63
Sleeping on the floor/ground	Yes	238	55.1
	No	194	44.9
Knows about Transmission of VL	Sandfly	131	30.3
	Mosquito	301	69.7
Knows about sign/symptoms of VL	Yes	95	22.0
	No	337	78.0
Knows about prevention of VL	Yes	210	48.6
	No	222	51.4

### Environmental, housing conditions, and animal ownership related factors

Among the study participants; 281 (65%), 252 (58.4%), 215 (49.8%), 235 (54.4%), 224 (51.9%), and 235 (54.4%) had animal shelter inside the compound, their house roof had a thatch construction, had cracked house walls, the presence of crack soil near the sleeping place, the presence of termite hills near the house, and the presence of at least one or more domestic animals near the sleeping place, respectively (Table 3).

**Table 3:** Environmental, housing condition, and animal related factors among study participants of Dire District, Borena zone, Oromia Region, Ethiopia, 2021 (*n* = 432).

**Characteristics**	**Categories**	**Number**	**Percent**
Presence of animal shelter inside the compound	Yes	281	65.0
	No	151	35.0
Types of House roof	Corrugated	180	41.6
	iron sheet		
	Thatch	252	58.4
Presence of cracked house wall	Yes	215	49.8
	No	217	50.2
Presence of crack soil near sleeping place	Yes	235	54.4
	No	197	45.6
Presence termites' hills near house	Yes	224	51.9
	No	208	48.1
Presence of water source/point near home	Yes	89	20.6
	No	343	89.4
Presence of Hyrax near home	Yes	45	10.4
	No	387	89.6
Presence of at least one or more domestic animals near sleeping place (Dog/cattle (goat and sheep)/donkey/cat)	Yes	235	54.4
	No	197	45.6

### Prevalence of visceral leishmaniasis

The overall seroprevalence of *L. donovani complex* was 33/432(7.6%) (95%CI: 5.32–15.60). This infection was found to be more prevalent in men [24/33(11.2%)], participants aged 10-to 19 years old 8/33(12.6%), those with cattle keeping occupations [23/33(12.2%)], a family monthly income of < 500 Ethiopian birrs [15/33(12.8%)], and a family size >5 [15/33(18.3%)]. It was relatively more prevalent in study participants from having a family history of infection with VL [13/33(15.0%)], living in house roof types of thatch [24/33(9.5%)], presence of hyrax near home [7/33(15.6%)], presence of water source/point near home [16/33(18.4%)] and presence of at least one or more domestic animals near sleeping place [24/33(10.2%)].

### Factors associated with seroprevalence of visceral leishmaniasis

In the bivariate logistic analysis variables, including sex, occupation, family monthly income, family size, travel history to other VL endemic areas, family history of infection with VL, sleeping or/and staying at night and/or day under the acacia tree, know about prevention of VL, presence of animal shelter inside the compound, presence of cracked house wall, presence of termite hills near the house, presence of water source/point near home and presence of at least one or more domestic animals near the sleeping place in the compound were significantly associated with seroprevalence of *Leishmania donovani complex* infection and considered as candidates for multivariable analysis (Table 4).

**Table 4:** Bivariable and multivariable logistic regression analysis factors associated with seroprevalence of visceral leishmaniasis in Dire District, Borena zone, Oromia Region, Ethiopia, 2021.

**Characteristics**	**Categories**	**Seroprevalence of** ***L. donovani***		**COR (95%CI)**	***P*-value**	**AOR**	***P*-value**
		**Reactive** **[No. (%)]**	**Non-reactive** **[No. (%)]**				
Sex	Male	24 (11.2%)	190 (88.8%)	2.902 (1.330–6.469)	0.008	1.750 (1.001–5.490)	0.422
	Female	9 (4.1%)	209 (95.9%)	1		1	
Age (in years)	1–9	4 (5%)	76 (95%)	1			
	10–19	8 (5.9)	128 (94.1%)	1.188 (1.524–5.408	0.381		
	20–29	11 (10.6%)	93 (89.4%)	1.981 (1.236–4.888)	0.446		
	≥30	10 (8.9%)	102 (91.1%)	1.863 (0.280–4.312)	0.687		
Occupation	Cattle keeping	23 (12.2%)	165 (87.8%)	3.095 (1.014–7.201)	0.023	1.095 (0.914–3.201)	0.141
	Students	5 (3.9%)	123 (96.1%)	0.902 (0.013–2.051)	0.595	0.016 (0.006–1.004)	0.758
	Trading/skilled job/Marchant	5 (4.3%)	111 (95.7%)	1		1	
Educational status	Illiterate	20 (9.4)	193 (90.6%)	2.021 (1.164–5.495)	0.312		
	Read and write	9 (6.6%)	128 (93.4%)	1.371 (1.230–3.448)	0.609		
	Elementary and above	4 (4.9%)	78 (95.1%)	1			
Family monthly income (in birr)	< 500	15 (12.8%	102 (87.2%)	3.015 (1.125–6.895)	0.029	2.513 (1.003–4.023)	0.015
	501–999	12 (6.5%)	173 (93.5%)	1.422 (0.257–1.925)	0.493	1.012 (0.408–2.346)	0.381
	≥1,000	6 (4.6%)	124 (96.1%)	1		1	
Family size	1–2	6 (3.7%)	156 (96.3%)	1		1	
	3–5	12 (6.4%)	176 (93.6%)	1.773 (0.207–1.539)	0.263	1.587 (0.121–4.848)	0.508
	>5	15 (18.3%)	67 (81.7%)	5.821 (2.064–7.462)	0.001	5.154 (2.032–9.748)	0.020
Travel history to other VL endemic kebele	Yes	26 (9.1%)	260 (90.9%)	1.986 (0.841–4.691)	0.112	0.602 (0.205–3.769	0.356
	No	7 (4.8%)	139 (95.2%)	1		1	
Family history of infection with VL	Yes	13 (15.0%)	74 (85.0%)	2.855 (1.359–5.998)	0.024	1.658 (0.221–6.959)	0.452
	No	20 (5.8%)	325 (94.2%)	1		1	
Use bed net	Yes	9 (10.3%)	78 (89.7%)	1		1	
	No	24 (7.0%)	321 (93.0%)	1.543 (0.690–3.471)	0.388		
Sleeping/staying at night or day under Acacia tree	Yes	22 (13.8%)	138 (86.2%)	3.783 (1.782–8.029)	0.001	2.984 (1.074–8.288)	0.036
	No	11 (4.0%)	261 (96.0%)	1		1	
Sleeping on the floor/ground	Yes	19 (7.9%)	219 (92.1%)	1.115 (0.544–2.287)	0.765		
	No	14 (7.2%)	180 (92.8%)				
Knows about prevention methods of VL	Yes	9 (4.3%)	201 (95.7%)	1		1	
	No	24 (10.8%)	198 (89.2%)	2.7071 (1.168–4.815)	0.011	0.226 (0.070–0.728)	0.315
Knows about sign/symptoms of VL	Yes	10 (7.4%)	85 (92.6%)	1			
	No	23 (7.7%)	314 (92.3%)	0.6226 (0.736–3.504)	0.370		
Knows about transmission of VL	Yes	8 (6.1%)	123 (93.9%)	1			
	No	25 (8.3%)	276 (91.7&)	1.393 (0.815–5.807)	0.446		
Presence of animal shelter inside the compound	Yes	20 (11.1%)	161 (88.9%)	2.274 (1.352–5.942)	0.032	1.821 (0.966–6.068)	0.421
	No	13 (5.2%)	238 (863%)	1			
Types of house roof	Corrugated iron sheet	11 (6%0	169 (94%)	1			
	Thatch	22 (8.7%)	230 (91.3%)	0.680 (0.321- 1.441)	0.327		
Presence of cracked house wall	Yes	22 (10%)	193 (90%)	2.135 (1.896–5.899)	0.022	1.801 (1.026–4.921)	0.033
	No	11 (5%)	206 (95%)	1		1	
Presence of crack soil near sleeping place	Yes	8 (3.4%)	227 (96.6%)	0.242 (0.042–2.378)	0.535		
	No	25 (12.7%)	172 (87.3%)	1			
Presence termites' hills near house	Yes	23 (10.3%)	201 (89.7%)	2.243 (1.103–4.896)	0.021	1.938 (1.002–7.050)	0.016
	No	10 (4.8%)	198 (95.2%)	1		1	
Presence of water source/point near home	Yes	16 (18.4%)	73 (81.6%)	4.203 (2.029–8.707)	0.001	3.983 (1.034–7.426)	0.001
	No	17 (4.3%)	326 (95.7%)	1		1	
Presence of Hyrax near the home	Yes	7 (15.6%)	38 (84.4%)	2.558 (1.470–4.260)	0.443		
	No	26 (7.0%)	361 (93.0%)	1			
Presence of at least one or more domestic animals near sleeping place (Dog/cattle (goat and sheep)/donkey/cat)	Yes	24 (10.2%)	211 (89.8%)	2.376 (1.228–4.939)	0.016	2.126 (1.341–5.108)	0.012
	No	9 (4.6%)	188 (95.4%)	1		1	

In multivariable logistic regression; < 500 Ethiopian Birr family monthly income, family size greater than five, sleeping/staying at night and/or day under the acacia tree, presence of cracked house wall, presence of termite hills near the house, presence of water/point source near home, and presence of at least one or more domestic animals near the sleeping place were statistically significant (Table 4).

Individuals with a family monthly income of < 500 Ethiopia Birr were almost 2.5 times (AOR: 2.513; 95%CI: 1.002–4.023) more likely to be infected with *Leishmania donovani complex* compared with individuals from a family in which the monthly income was ≥1,000 Ethiopia Birr. Individuals from a family of more than five people were 5.1 times more likely (AOR: 5.154; 95%CI: 2.032–9.748) to develop infection compared to households with a family size that was less or equal to two. Participants being sleeper/stayed at night or/and day under an acacia tree were almost 3 times (AOR: 2.984; 95%CL: 1.074–8.288) more likely to acquire VL infection compared with their counterparts (Table 4).

In terms of being more likely to develop VL infection compared, the odds of the presence of a cracked house wall meant people were 1.8 times more likely to contract it (AOR: 1.801; 95%CI:1.026–4.921). Having a termite hill near the house meant people were almost 2 times more likely (AOR: 1.938; 95%CI:1.002–7.050). Water sources/points near the home meant that participants were 4 times more likely (AOR: 3.983; 95%CI:1.034–7.426), and the presence of at least one or more domestic animals near the sleeping place meant people were 2.126 times more likely to contract infection (AOR: 2.126; 95%CI: 1.341–5.108) (Table 4).

## Discussion

This study was undertaken to assess the seroprevalence of VL and its associated factors among the asymptomatic pastoral community of the Dire District in the Borena Zone of the Oromia Region, Ethiopia. The overall seroprevalence of *the Leishmania donovani complex* was 7.6% (95%CI: 5.32–15.60). Risk factors that aggravate the occurrence of VL infection include earning < 500 Ethiopian Birr for the monthly family income, having a family size greater than five, sleeping/staying at night and/or day under an acacia tree, the presence of cracked house walls, the presence of termite hills near the house, the presence of water points or sources near the home, and the presence of at least one or more domestic animals near the sleeping place.

In the current study, the overall seroprevalence of *L. donovani complex* was 7.6%. Similar results are reported from studies conducted in Benishangul Gumz (6.9%) (28), Tigray (8.8%) (29), the Gode district of Shebel zone of the Somali region (12.7%) (30), and the west Armachiho district (7.6%) (26). However, these results were higher than those reported in previous studies conducted in the Benishangul-Gumz (3.2%) (25) and Raya Azebo district of northeastern Ethiopia (0.87%) (31). However, they were less when compared to reports from Adadile district (31.1%) (30), Addis Zemen North West Ethiopia (39.1%) (22), and SNNPR (36.0.4%) (32). These differences might be due to the varied implementation of different intervention strategies, settlement of the community, and the different study population and design, as well as factors such as the socioeconomic status of the area and living conditions of the communities studied.

In the current study, a family size of more than 5 members in the household had a statistically significant association with the seroprevalence of VL infection. This study finding was consistent with those conducted in northwestern Ethiopia (22) and north-central Ethiopia (33). The higher amount of carbon dioxide and odors released in the homestead could attract sandflies and increase the chance of asymptomatic cases.

In this study, a low family monthly income was a statistically significant association, with seroprevalence of VL compared to their counterparts. These results were contrary to the studies conducted in the Pokot territory of Kenya and Uganda (34). In previous studies, living in a straw-roofed house was the house characteristic most associated with asymptomatic infection. This could be related to socioeconomic status or to the potential that straw roofs may provide resting places for sand flies and increase their survival in the homestead area (35). The housing condition and overall environment are favorable for breeding and resting sites of sandflies.

According to a study conducted in Kenya and Uganda, poor nutritional status has been associated with a higher risk of developing VL (36). In the present study, there was a strong association between the seroprevalence of *L. donovani complex* and the presence of poor housing conditions such as house wall cracks. This finding is supported by a study by Yared et al. that confirmed individuals who sleep regularly near cracked walls are at higher risk of VL infection (22). The possible reason for this might be that cracked walls are ideal breeding and resting sites for sand flies. Furthermore, mud-type houses have been identified as risk factors for asymptomatic VL infection as sand flies prefer mud crack walls for breeding and resting sites (37).

The findings of the current study show that sleeping or/and staying day or/and night under an acacia tree was significantly associated with seroprevalence of *L. donovani complex*. This is consistent with a previous study carried out in the Shebele zone of the Somali region (30) and the Gedaref state of Sudan (24). This might be due to the sand flies using the acacia vegetation tree as breeding/resting sites and having access to bite people while they sleep or stay under the tree. Another study from Ethiopia conducted in Libo Kemkem and Fogera, also reported that resting under acacia trees was a risk factor for VL (8) as acacia vegetation provides suitable breeding/resting sites for Phlebotomine sand flies (8, 38).

In this study, the presence of water points/sources near the home increased the likelihood of VL infection by 3 fold. This finding was supported by studies in Tigray Northern Ethiopia (39), and in north-central Ethiopia (40). In our study, more than 50% of the seroprevalence of the *L. donovani complex* was found in participants from Magado Kebele. The reason for the increment of cases in this area might be related to the settlement of the community near the water point of Ele_Bora. This may create favorable conditions for the breeding of sandflies. Moreover, all the cases from other kebele had a travel history that involved going to the water point in the study area.

In the current study, the presence of a domestic animal in the yard had a statistically significant association with the seroprevalence of *L. donovani* complex infection. The majority of the *Leishmania* species are involved in zoonotic transmission. Infected animal reservoir hosts transmitted pathogens to the human population and spillover events result in zoonotic diseases (41). This finding was supplemented by the study conducted in Armachiho district (26) and Shebel zone, Somali region, Ethiopia (30). The presence of a domestic animal in the yard increases the chance of getting an infection might be due to the zoonotic nature of the disease (42). Some of the domestic animals may attract vectors to homesteads and increase the probability of human exposure to sandflies. In the study area, the communities are pastoralists, and the presence of domestic animal shelters in their compounds may attract sand fly vectors to breed and rest there. In the current study; the presence of livestock and their manure in the compound could be a favorable breeding area for the sand fly's vectors.

The present study revealed that the presence of termite hills near houses had a statistically significant association with an increase of leishmaniasis infections. This is supported by the study conducted in the Pokot territory of Kenya and Uganda (34), the Shebele zone of the Somali region (30), and Armachiho district, Ethiopia (26). The termite's hill represents ideal conditions, providing breeding and resting sites for sandflies. The findings of this study might due to the majority of the landmarks being covered by the termite hills.

The presence of cracked soil was not significant. This is supported by a study conducted in Gedaref state, Sudan (24). This study is contrary to that study done in the Armachiho district (26) and Tigray regions in northern Ethiopia (22). The reason for these differences might be that the majority of the land is covered by sandy soil, which fills the cracked soil and may not be favorable for sandflies.

In the present study, utilization of bed net was not a statistically significant association with the seroprevalence of VL. On the contrary, a study conducted in Dessie town north-central Ethiopia (40) reported a statistically significant association. The reason for this discrepancy might be that access to and utilization of bed nets in the study area were very low, and only 18% of people had a bed net.

A travel history of visiting other endemic areas was not significantly associated with the seroprevalence of VL. This is contrary to a study conducted in the Somali region in southern Ethiopia (43) and Dessie town in north central Ethiopia (40). The possible reason for these differences might be that the community of the study area were pastoralists and they did not typically travel to other endemic areas, but there was an area that acted as a source of infection, the so-called Ele-Bora water source used for cattle.

The strength of this study was that it examined a hard-to-reach area. It determined the seroprevalence of VL infection at the asymptomatic pastoralist community level using both structured questionnaires and serological tests. The limitations of this study are that the laboratory methods used could not be rolled out past the current infection. Since this was a single snapshot measurement of exposure and outcome, it is difficult to derive causal relationships from the cross-sectional study.

In Ethiopia, despite a significant number of people being at risk of contracting VL, the zoonotic nature of the disease, and the spread of the disease to non-endemic areas due to environmental and climate changes, there is no active and routine screening of leishmaniasis cases. Thus, the health sectors and concerned bodies need to create tailored programs and intervention packages to make the community more aware and enable them to prevent the transmission and control of the disease, increasing the diagnosis, screening, and surveillance of VL using different laboratory-based approaches is essential for providing up-to-date evidence and design policy to control disease in the endemic area and at the national level.

## Conclusion

The seroprevalence of VL among the asymptomatic pastoralist community was comparable with the majority of previous studies. The main factors associated with the seroprevalence of VL infection were sleeping or/and staying day or/and night under an acacia tree, an increased family size of more than five members, the presence of cracked house walls and termite hills, and the presence of water sources/points and domestic animals near the home. Awareness of VL transmission and prevention in pastoral communities and taking appropriate interventions, and focusing on factors that aggravate VL infection are recommended for preventing and controlling disease transmission in the area. Further comprehensive studies on the epidemiology and magnitude of the disease, and investigations of other contributing risk factors in the area using advanced methods and laboratory investigations are required.

## Data availability statement

The original contributions presented in the study are included in the article/supplementary material, further inquiries can be directed to the corresponding author.

## Ethics statement

The studies involving human participants were reviewed and approved by Haramaya University, College of Health and Medical Sciences Institutional Health Research Ethics Review Committee (IHRERC). Written informed consent to participate in this study was provided by the participants' legal guardian/next of kin.

## Author contributions

HK undertook the data collection and the study idea. HK, FW, AG, and TG designed the manuscript, interpreted the results and drafted the manuscript, and including tables and figures. All authors critically reviewed the manuscript and approved the final submitted version.

## Funding

Haramaya University postgraduate directorate covered financial support for research data collection.

## Conflict of interest

The authors declare that the research was conducted in the absence of any commercial or financial relationships that could be construed as a potential conflict of interest.

## Publisher's note

All claims expressed in this article are solely those of the authors and do not necessarily represent those of their affiliated organizations, or those of the publisher, the editors and the reviewers. Any product that may be evaluated in this article, or claim that may be made by its manufacturer, is not guaranteed or endorsed by the publisher.
